# The Role of Primary Health Care in Hepatitis B Testing and Management: A Case Study

**DOI:** 10.1007/s10900-017-0385-9

**Published:** 2017-06-22

**Authors:** Jacqueline A. Richmond, Joe Sasadeusz, Meredith Temple-Smith

**Affiliations:** 1 The Royal Melbourne Hospital, Victorian Infectious Diseases Service, 300 Grattan Street, Parkville, Melbourne, 3000 Australia; 2 Viral Hepatitis Research Program, Australian Research Centre in Sex, Health & Society, La Trobe University, 215 Franklin Street, Melbourne, 3000 Australia; 3 Department of General Practice, Faculty of Medicine, Dentistry and Health Sciences, The University of Melbourne, 300 Barry Street, Parkville, Melbourne, 3000 Australia; 4 Department of General Practice, Faculty of Medicine, Dentistry and Health Sciences, The University of Melbourne, 200 Berkely Street, Parkville, Melbourne, 3000 Australia

**Keywords:** Hepatitis B, Testing, Diagnosis, Primary health care, Quality improvement

## Abstract

Hepatitis B is a complex disease requiring lifelong management. Infection is linked to birth in high prevalence regions including Africa and Asia. Best practice guidelines identify who to test for hepatitis B, however, a significant proportion of Australians with hepatitis B have not been diagnosed, and are subsequently at risk of serious morbidity and mortality. This study sought to address the gap between current and optimal hepatitis B testing in a primary care clinic with a likely high population of undiagnosed hepatitis B. Between September 2015 and December 2016, four interventions aimed at enhancing general practitioner testing practices were implemented: staff education, quality improvement and patient-triggered activities. Compared to the baseline (2014) the following parameters all increased in 2016: the number of patients tested (15 tests per month in 2014, 24 tests per months in 2016), the correct ordering of the recommended tests (17% in 2014, 61% in 2016) and hepatitis B vaccine dose ordering (n = 35 in 2014, n = 110 in 2016). However, the proportion of patients born in Africa or Asia tested for hepatitis B did not increase. Distribution of a patient held-reminder led to the greatest number of tests being ordered (n = 54 tests ordered in 1 month). Within a single primary care clinic situated in a high hepatitis B prevalence area, an intervention designed to improve adherence to hepatitis B testing guidelines, increased testing levels. A systematic approach can assist general practitioners to improve their understanding of hepatitis B testing and prioritise people most at risk.

## Introduction

Globally, 400 million people have Chronic Hepatitis B (CHB) with the most significant burden experienced in Asia and Africa. The predominant routes of transmission are mother-to-child and sexual contact [1]. Best practice guidelines demonstrate that if CHB is diagnosed early, and managed and treated appropriately the risk of death related to hepatocellular carcinoma (HCC) and liver disease is significantly reduced [2–4]. In Australia, 239,167 people are estimated to have CHB, however, low diagnosis rates, low uptake of both specialist management and antiviral treatment and late diagnosis of hepatitis B related-HCC mean that hepatitis B is a cause of significant morbidity and mortality [5, 6].

A goal of 80% diagnosis rate by 2017 was set in the Australian National Hepatitis B Strategy 2014–2017 [7]. Although the Australian Hepatitis B Testing Policy [8] recommends hepatitis B testing for all people born in intermediate or high prevalence countries; Aboriginal or Torres Strait Islander people; children of women who have CHB; unvaccinated adults at high risk of infection; people with a family history of chronic liver disease or HCC or abnormal liver function tests or acute hepatitis; and family, sexual or household contacts of a person with or suspected to have hepatitis B, current estimates indicate that only 38% of people with hepatitis B have being diagnosed [5].

Given their focus on identification of risk factors, screening and vaccination programs and opportunity to support life-long monitoring, the role of general practitioners (GPs) in managing CHB has been explored as an alternative to specialist-led care [9]. However, numerous studies have shown that GPs’ hepatitis B knowledge is poor, and many are unsure about who or how to test, or understand the link between CHB and HCC [10–12]. General practitioners’ lack of understanding of hepatitis B also negatively affects patients’ understanding of their infection and adherence to clinical management. Considering this, interventions focused on improving GP practice of hepatitis B testing, monitoring and management are needed to address the gap between best practice and the current sub-optimal management strategies.

Financial incentives, and audit and feedback on performance are two interventions commonly used to motivate GPs to undertake specific healthcare activities [13]. Other interventions designed to improve adherence to screening guidelines include linking screening to routine testing, capacity building activities including education, development of performance indicators and the use of prompts in electronic medical records (EMR) [14–16]. Such clinical interventions are most successful when they are shown to be effective, capable of being widely implemented and can be normalised into routine practice [17].

This case study explored the impact of four different interventions which sought to improve rates of hepatitis B testing in one primary care clinic over a 15 month period.

## Methods

The study was conducted between September 2015 and December 2016. The primary care clinic is located in inner metropolitan Melbourne in the tenth highest prevalence area for hepatitis B in Australia [5]. The clinic employs 11 GPs and three nurses, and had a patient caseload of approximately 3000 in 2016.

A memorandum of understanding was developed between the study partners to outline the expectations and responsibilities of each party in relation to the provision of hepatitis B care and capacity building activities at the primary health care clinic. An Advisory Committee of stakeholders, comprising three clinic staff (GP, lead nurse and practice manager), an infectious diseases specialist physician, a tertiary hospital based viral hepatitis nurse and a community-based organisation representative, met quarterly during the project and advised on the design, implementation and evaluation of the interventions. Ethics approval was obtained from the La Trobe University Human Ethics Committee and the primary care clinic Human Ethics Advisory Group. Access to patients’ EMR for the purpose of conducting quality assurance activities was also specifically addressed in the memorandum of understanding.

A group discussion was conducted with the clinic GPs and nurses in June 2015 during the design phase of the study, to focus the study aims and outcomes and to explore GP preferences for interventions. The discussion was framed around the Normalisation process theory [18], which states that for a complex intervention to be successfully normalised, four criteria must be met: all staff must agree that there is a problem to be addressed, and that the intervention will assist in addressing the problem. All staff must be aware of their role in the intervention, and agree on a way to deal with problems as they arise.

### Patient Population

A baseline audit of the EMR was conducted in June 2015, using the 14 codes available for hepatitis B in the medical practice software to determine the number of patients with CHB, at-risk of hepatitis B, tested and vaccinated for hepatitis B. An active patient was defined as having attended the clinic since 1st January 2010; no age limits were applied to the source population.

### Interventions

The project involved the implementation of four interventions during the study period: education, audit and feedback cycle, review of EMR and patient held reminder (Table 1).

**Table 1:** Content, target group and implementation timeframe of the four interventions of this study

Intervention	Content of intervention	Target group	Time frame for implementation of the intervention
Education	Case-based education delivered by hepatitis B GP champion external to the clinic and researcher (JR)	GPs*, clinic nurses, allied health professional and administrative staff Patients attending the clinic	September to November 2015: Three case-based GP education sessions were offered on different days to accommodate the part time GP workforce Nursing, allied health and administrative education was provided monthly over three months Consumer education was provided through existing patient advisory committees and poster and resource display in the clinic waiting room
Audit and feedback cycle	The audit data was fed back at team meetings using “gamification” as a report back strategy.^a^ “Gamification” involves applying elements associated with game playing such as point scoring or competition with others. In this project, gamification was used to engage GPs in a competition to increase the number of hepatitis B tests they had ordered in the previous audit cycle Individual data about their own testing behaviour was also provided to each GP via electronic mail	GPs	Repeated every 3 months during the project for a total of five times: December 2015, March, July, October, December 2016
Review of Electronic Medical Record (EMR)	Review of each patient’s EMR to assess risk factors for hepatitis B infection (country of birth, household contact, and risk behaviour such as injecting drug use or high risk sexual activity), previous hepatitis B testing and or vaccination status An EMR prompt was inserted for the GP to follow-up, which included testing or information to encourage vaccination	GPs	EMR reviews were conducted on every patient who had a medical appointment during the first week of February, April and June 2016
Patient-held reminder card	The administration staff provided the card to the patient at registration. Patients were instructed to hand the card to the doctor at the beginning of the consultation	GPs	The patient-held reminder card was distributed in May and September 2016

*The practice manager quarantined the education time so GPs could attend

^a^Fox J. The game changer: how to use the science of motivation with the power of game design to shift behaviour, shape culture and make clever happen. Milton, Queensland Wiley, 2014

### Outcome Measures

Outcome measures were:

- The number of previously untested patients tested for hepatitis B.
- The number of patients who were correctly tested for HBsAg, anti-HBc, and anti-HBs.
- The proportion of patients from priority populations (born in Asia or Africa and people who identify as Aboriginal or Torres Strait Islander) tested for hepatitis B.
- The number of hepatitis B vaccine doses administered.

### Data Analysis

The number of patients tested for hepatitis B was provided in an Excel spreadsheet on a quarterly basis by the onsite pathology provider. This data set was further developed during the audit and feedback cycle of the project. The EMR of each patient tested for hepatitis B during 2014, 2015 and 2016 was reviewed by the researcher +/− a clinic staff member, and consensus was reached on an assessment of their case definition and subsequent management. Data frequencies were calculated using pivot tables in the Excel spreadsheet.

### Clinic Characteristics

In 2016, 11 GPs were employed at the clinic, ten were female, and the majority were employed part time (Table 2). The majority of patients attending the clinic were born in Australia (54%), 29.3% were born in intermediate hepatitis B prevalence countries and the remaining born in high hepatitis B prevalence countries including Greece (4.5%), Italy (3.5%), Vietnam (3.5%), Somalia (2.7%) and Ethiopia (2.5%).

**Table 2:** Effective full time equivalence for the general practitioners working in the clinic in 2016

Effective full time (EFT)	Hours per week (h)	Number of general practitioners
0.1–0.3	6–13	6
0.5	20	1
0.71	27	2
1.0	38	2

## R**e**sults

The following section describes the impact of the four interventions on the hepatitis B testing practices of the clinic GPs.

At the beginning of the project in 2014, an estimate of the number of patients with CHB attending the clinic was calculated based on the hepatitis B prevalence of the patients’ country of birth [19]. By this method it was estimated there were 100 patients with CHB attending the clinic. However, the initial audit of the EMR identified only 13 patients with CHB, suggesting a significant shortfall in diagnosis.

The group discussion conducted during the design phase involved four GPs and three clinic nurses, and revealed a low level of knowledge and confidence in CHB testing and management. A request for small group education led to the first intervention. Nine of 15 GPs attended one education session; four GPs attended all three sessions. Six GPs did not attend any of the education sessions as they did not work at the clinic on the days the education sessions were offered. The researcher provided tailored education to three of the six GPs during individual consultations. The remaining three GPs only worked on Saturdays and were not interested in receiving education.

### Hepatitis B Testing Practices

**Graph 1:**
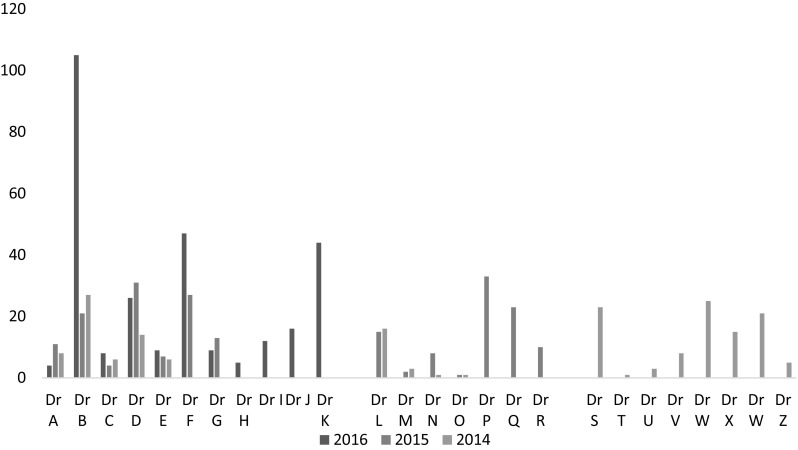
Hepatitis B tests ordered by individual general practitioners in 2014, 2015 and 2016

Overall there was an increase in the number of tests ordered between 2014 and 2016. The average number of tests ordered by GPs per month increased from 15 in 2014 to 24 in 2016. The testing practices of each GP is presented in Graph 1. Of the 26 GPs who worked at the clinic between 2014 and 2016, only five (Drs A to E) were employed over the triennium. Doctors B, F and K ordered the highest number of hepatitis B tests in 2016; each attended the three educational sessions offered during Intervention one and participated in the medical meetings when the audit data was presented.

Table 3 outlines the type and timing of interventions and the corresponding number of tests ordered. The highest number of tests were ordered in May 2016 during Intervention four.

**Table 3:** Number of hepatitis B tests ordered by intervention and month

Month and year	Intervention	Total number of tests ordered per month
Sept-15	Education	34
Oct-15	Education	30
Nov-15	Education	17
Dec-15	Audit and Feedback	14
Jan-16	No activity	10
Feb-16	Electronic medical record (EMR) review and prompt	18
Mar-16	Audit and feedback	25
Apr-16	EMR review and prompt	22
May-16	Patient held reminder	54
Jun-16	EMR review and prompt	27
Jul-16	Audit and feedback	20
Aug-16	No activity	20
Sep-16	Patient held reminder	22
Oct-16	Audit and feedback	30
Nov-16	No activity	22
Dec-16	Audit and Feedback	15
Total number of hepatitis B tests ordered during the intervention period (September 2015 to December 2016)		380

In order to gain a comprehensive understanding of patients’ hepatitis B status and susceptibility to infection the Australian Hepatitis B Testing Policy [8] recommends that hepatitis B testing should include three tests: HBsAg, anti-HBs and anti-HBc. Table 4 outlines the GP testing patterns between 2014 and 2016. The proportion of times the three tests were correctly ordered increased substantially between 2014 (17%) and 2016 (61%). This activity was a focus of Interventions one, two and three.

**Table 4:** Testing patterns of the general practitioners in 2014, 2015 and 2016

Year and number of patients tested for hepatitis B	Three hepatitis B tests ordered (HBsAg, anti-HBc, anti-HBs)	Two tests ordered	One test ordered
2014 (n=183)	17% (31)	72% (132)	11% (20)
2015 (n=206)	40% (82)	56% (115)	4% (9)
2016 (n=285)	61% (174)	34% (96)	5% (15)

### Hepatitis B Testing According to Country of Birth

While the overall number of patients tested for hepatitis B increased between 2014 and 2016, the proportion of patients born in Africa and Asia did not increase (Table 5). There was no difference between 2014, 2015 and 2016 in the proportion of people tested according to country of birth with most patients tested for hepatitis B in 2016 being born in Australia.

**Table 5:** Comparison of demographic characteristics of patients tested for hepatitis B in 2014, 2015 and 2016

Year the hepatitis B test was ordered	2014	2015	2016
Number of hepatitis B tests ordered	183	206	285
Mean age of patients tested	34 years	41 years	43 years
% females	67% (123)	63% (130)	56% (161)
Number of antenatal hepatitis B tests ordered	11% (21)	9% (18)	10% (28)
% Continent of birth^a^			
Africa	20% (37)	22% (45)	19% (55)
Asia	6% (11)	9% (18)	12% (34)
Australia and Oceania	37% (67)	47% (96)	44% (125)
Aboriginal and Torres Strait Islander	5% (9)	1% (3)	2% (7)
Europe	8% (15)	8% (17)	13% (36)
North America	(0)	0.5% (1)	1% (2)
South America	(0)	0.5% (1)	0 (0)
Missing data	24% (44)	12% (25)	9% (26)

^a^ http://www.countries-ofthe-world.com

The country of birth of all patients attending the clinic in 2016 is listed in Table 6, along with the breakdown of individual countries in the highest hepatitis B prevalent regions. Patients born in Ethiopia and Somalia were more likely to be tested for hepatitis B than patients born in other high prevalence countries. This finding may be an artefact of antenatal screening which includes hepatitis B testing. Of the 28 women tested during antenatal screening, 11 (39%) were born in Africa.

**Table 6:** Country of birth for patients attending the clinic in 2016 and proportion of patients tested for hepatitis B by country of birth

Country of birth for patients attending the clinic* (n=2994)	% (n) of patients attending the clinic according to country of birth	% (n) of patients tested for hepatitis B in 2016 according to country of birth
Australia	54% (1613)	42% (120)
China	3.2% (95)	1% (2)
Egypt	1.2% (37)	0.5% (1)
England	2.2% (66)	2% (5)
Eritrea	1.2% (35)	2% (7)
Ethiopia	2.5% (75)	8% (24)
Greece	4.6% (137)	5% (15)
Italy	3.6% (107)	1% (3)
Malaysia	1.0% (30)	0.5% (1)
New Zealand	1.5% (46)	1% (2)
Somalia	2.7% (81)	5% (13)
Sudan	1.4% (43)	3% (8)
Turkey	1.1% (33)	1% (4)
Viet Nam	3.3% (100)	5% (14)
Missing/not stated	3.3% (99)	9% (26)

*Only countries of birth with >1% of the total population of patients attending the clinic in 2016 are included; therefore total proportion does not equal 100%

### Hepatitis B Test Results and Management

**Graph 2:**
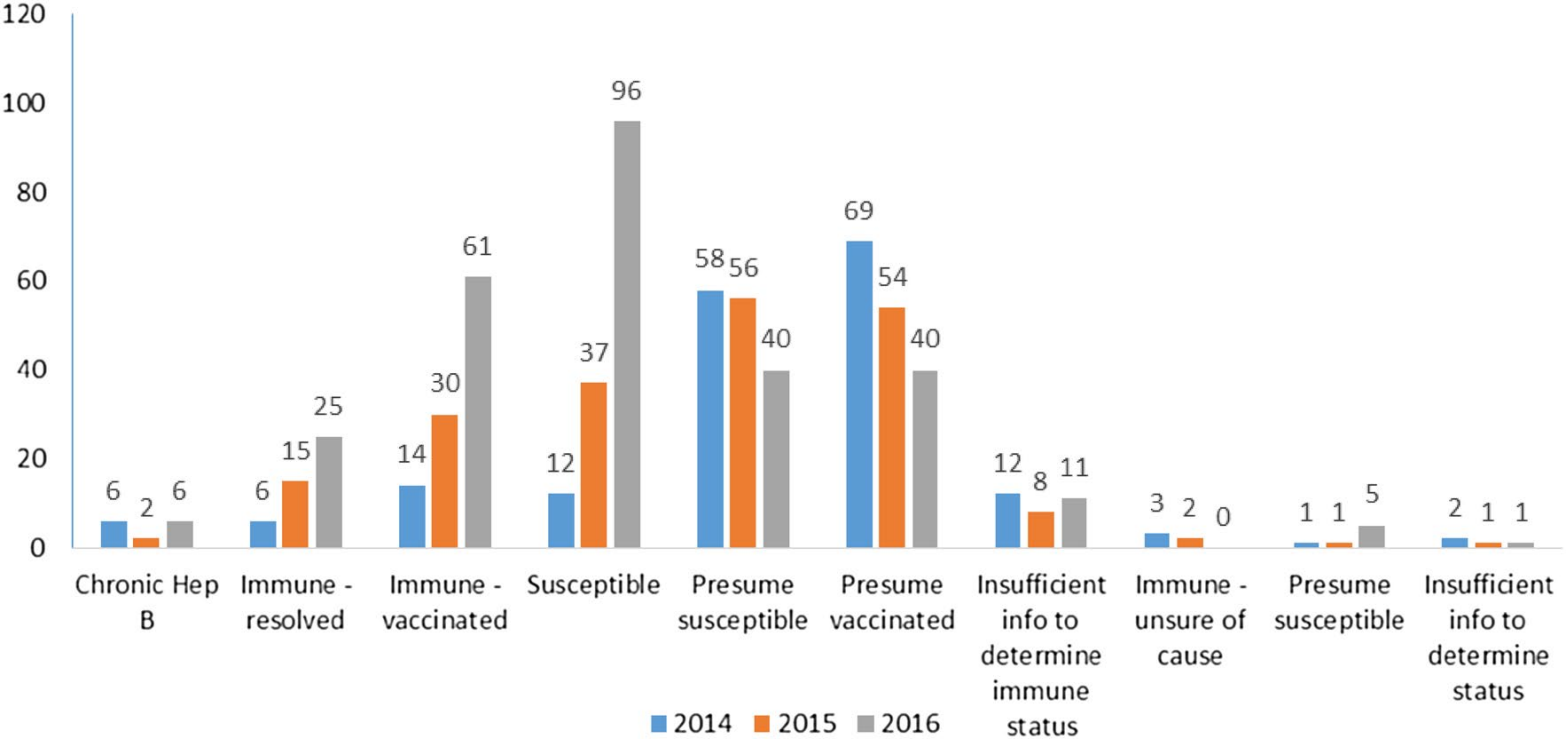
Results of hepatitis B tests in 2014, 2015 and 2016

The case definitions for the hepatitis B test results are provided in Table 7. The hepatitis B test results indicate the number of patients presenting to the clinic and susceptible to hepatitis B infection increased between 2014 (n = 12) and 2016 (n = 96) (Graph 2). This may be related to the increase in correct ordering of the three tests and subsequent ability to comprehensively assess patients’ immunity and susceptibility. Six HBsAg positive patients were identified in 2016, however three had previously been diagnosed and were re-tested during antenatal screening.

**Table 7:** Case definitions for hepatitis B test results

Case definition	Pathology results
Chronic hepatitis B	HBsAg detected and/or HBV viral load detected
Immune—resolved	Anti-HBs detected Anti-HBc detected
Immune—vaccination	Anti-HBs detected (HBsAg and anti-HBc not detected)
Susceptible	HBsAg not detected Anti-HBs not detected Anti-HBc not detected
CHB negative, presume susceptible to infection	HBsAg not detected Anti-HBs not detected Anti-HBc unknown
CHB negative, presume vaccinated	HBsAg not detected Anti-HBs detected Anti-HBc unknown
CHB negative, insufficient information to determine immune status	HBsAg not detected Anti-HBs unknown Anti-HBc unknown
Immune—vaccine-derived immunity or Natural immunity	Anti-HBs detected HBsAg unknown Anti-HBc unknown
Presume susceptible to infection	Anti-HBs not detected HBsAg unknown Anti-HBc unknown
Insufficient information to determine status	Anti-HBc detected or not detected Anti-HBs unknown HBsAg unknown

**Graph 3:**
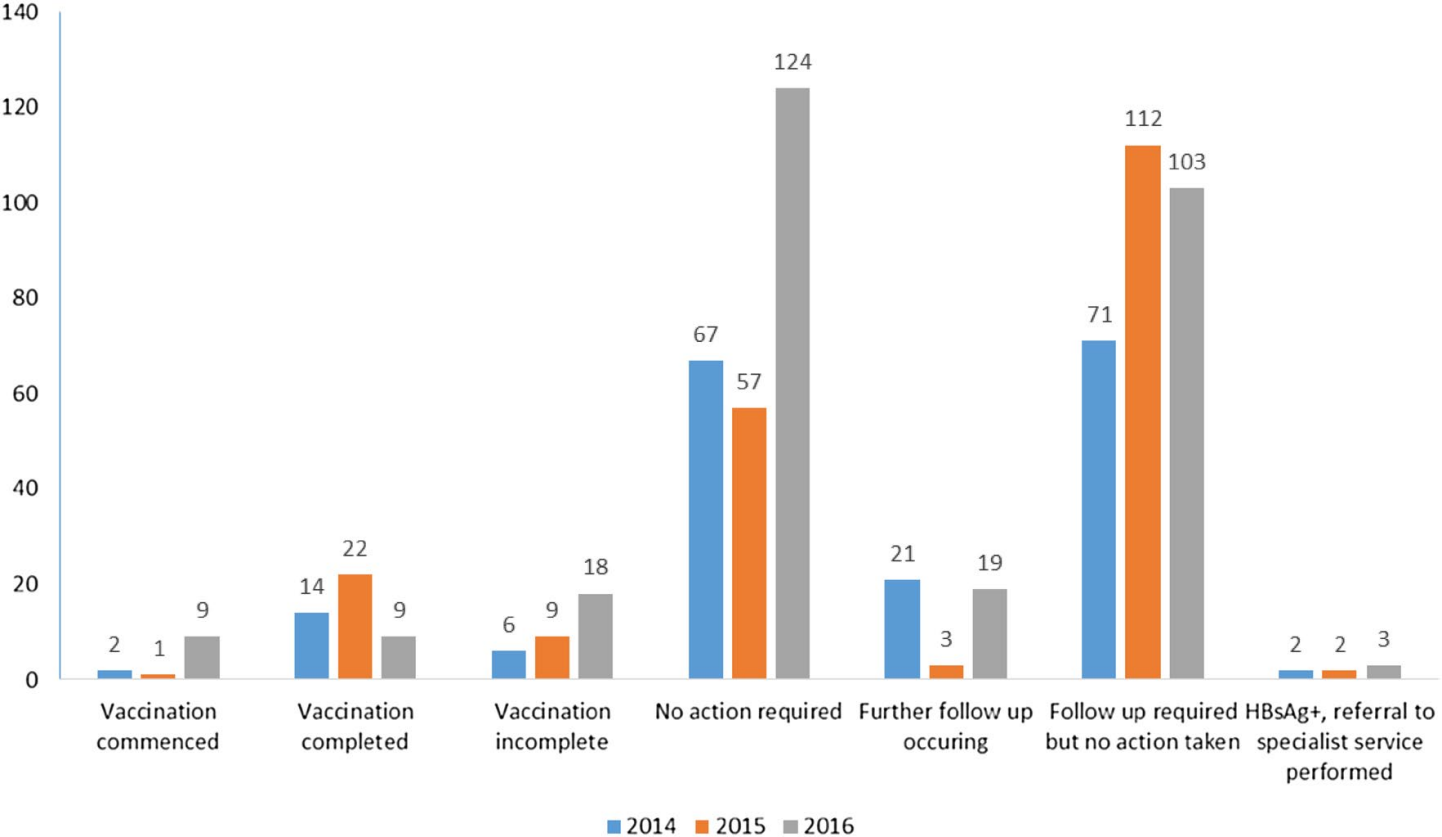
Medical management strategy provided after hepatitis B testing 2014, 2015 and 2016

Subsequent management of patients tested for hepatitis B was inconsistent (Graph 3). In 2016, 124 patients were tested and no further action was required because they had either been vaccinated or had resolved infection. One hundred and three patients required follow up including further hepatitis B tests (where only one or two tests had been ordered) or vaccination because they were susceptible, but no further action was documented in the patient’s EMR. The number of patients with incomplete vaccination status (only one or two of the three doses administered) increased over the 3 years from six in 2014 to 18 in 2016.

The number of hepatitis B vaccine doses ordered between 2015 and 2016 increased. In 2016, 110 doses were ordered compared to 35 in 2014. Tracking the number of vaccine doses administered was difficult because of incomplete documentation in the EMR.

## Discussion

Australia is considered a low hepatitis B prevalence country, however its cultural diversity means there are localised areas of intermediate to high prevalence. Given the projected high hepatitis B prevalence of the case study site and cultural diversity of its residents, the low number of patients identified with CHB attending the clinic was an unexpected finding. Based on the available data it was assumed there was an evidence-practice gap related to implementation of the Australian Hepatitis B Testing Policy. However, even with increased testing the number of patients identified with CHB did not increase. The case study results suggests that local epidemiological modelling may not be translatable to this primary care setting.

General practice can be a very unstable environment with GPs and patients moving in and out of the system. In this one clinic, only four GPs worked regularly between 2014 and 2016 and it was a predominantly part time workforce. Compared to 2014, the number of tests ordered in 2016 increased, however, the increase does not appear to have been sustained as reflected in a reduction to pre-intervention levels during periods of no activity. It is difficult to determine whether the knowledge that their testing practices were being monitored (Hawthorne effect) [20] led to a modification in their testing behaviour. While it is difficult to identify the impact of individual interventions on testing behaviour, because they ran concurrently, several positive practices were identified including the correct ordering of the serology tests (HBsAg, anti-HBc, and anti-HBs) and an increase in hepatitis B vaccine ordering. Correctly ordering hepatitis B tests reduces the barriers of repeat blood tests and allows for an immediate and comprehensive assessment of the patient’s status, including need for vaccination; an outcome which also increased in this case study.

Improvements in testing behaviour were not translated to the provision of follow up care. In 2016, 96 patients (34%) were identified as susceptible to hepatitis B infection; neither vaccinated nor infected. However, the proportion of patients who did not receive follow up care remained high (37%) in 2016. Inadequate follow up was also identified in the context of immunisation. Of the 18 patients who had not completed the three dose immunisation in 2016, 15 had received two of the three doses. While it is challenging to encourage patients to return for a third dose (6 months later), it is an example of a missed opportunity for the nursing staff to use electronic reminders in the practice software.

General practitioners are accustomed to testing women for hepatitis B during antenatal screening. The proportion of women screened during antenatal care remained consistent over the 3 years, with many identified as being susceptible to infection, but follow up vaccination was only initiated in two cases post-partum. It is possible that interventions implemented during the project were focused on testing and did not provide GPs with enough support and guidance on follow up care. Another possible explanation is the widespread screening strategy adopted by several GPs within the clinic, meant that hepatitis B tests were added to existing pathology requests, with no established plan to follow up the results.

Country or continent of birth, primarily Africa and Asian countries, were promoted as the primary risk factor to trigger testing. Unfortunately the proportion of patients born in Africa and Asia tested for hepatitis B did not change between 2014 and 2016. This may be explained by variability in the testing behaviour of individual GPs. Several GPs expressed discomfort in ‘racially profiling’ their patients, instead preferring to test all patients, which led to the popularity of Intervention four (patient held reminder), which had the greatest impact on tests ordered. Anecdotal reports indicated the reminder card allowed the patient to initiate a conversation about hepatitis B testing, instead of the GP. It is possible one of the barriers to testing may have been that GPs lacked a ‘script’ for introducing hepatitis B into the patient consultation which has been reported in other clinical areas [21]. Patient-initiated testing, as occurred during intervention four, allowed GPs to prioritise testing from the complex range of health issues most patients attended with.

Interventions that aim to increase GP performance need to be combined with patient education strategies. While information was displayed in the clinic waiting room, it was difficult to evaluate the impact. Several large-scale initiatives in the United States of America, Asia Pacific, and Europe demonstrated the feasibility of community-based interventions in effectively screening large numbers of people with CHB [22]. However, limited resources mean this is not always possible. Anecdotally in this case study, the perceived stigma associated with hepatitis B among patients from Asia and Africa [23] created barriers to patient engagement. However, the most successful intervention involved patients requesting a test through the patient-held reminder.

There are several limitations to this single-site, case study related to the complexity of the concurrent implementation of the interventions; it is impossible to confidently identify which intervention had the greatest impact. A significant learning from this case study is the need to engage GPs at the outset of any interventional project to confirm that the topic and methodology is clinically and contextually appropriate. The GPs in this clinic were not consulted about their interest in participating in this project prior to its commencement, as the memorandum of understanding was negotiated with the clinic management. While the researcher attempted to include the GPs in the design phase, the GPs should have been consulted about their clinical priorities before the proposal was accepted. Lack of interest and priority as well as GP fatigue regarding hepatitis B were noted during the project. Of interest, higher rates of testing did not increase the identification of patients with CHB. Hepatitis B was not a priority health condition for these GPs because they were caring for very few patients with CHB.

In Australia, there is an attempt to shift the care of patients with CHB from tertiary to primary care [7]. Initiating hepatitis B testing in primary care is critical to reducing the morbidity and mortality of undiagnosed infection. It is clear from this case study this will only be achieved if GPs are engaged and prioritise hepatitis B as an issue in their patient cohort which leads to the adoption of hepatitis B testing as a standardised clinical practice. Normalisation process theory states that changing GP behaviour requires a shared understanding and commitment by all staff to achieve the intended outcomes. Consultation with primary care practitioners about their clinical priorities must be the first stage in identifying evidence-practice gaps. Increases in hepatitis B testing are unlikely to occur in general practice without a coordinated and systematic approach.
